# A stochastic generative model for citation networks among academic papers

**DOI:** 10.1371/journal.pone.0269845

**Published:** 2022-06-29

**Authors:** Yuichiro Yasui, Junji Nakano

**Affiliations:** 1 Department of Statistical Science, School of Multidisciplinary Sciences, The Graduate University for Advanced Studies, SOKENDAI, Tokyo, Japan; 2 Department of Global Management, Chuo University, Tokyo, Japan; Xinjiang Technical Institute of Physics and Chemistry, Chinese Academy of Sciences, CHINA

## Abstract

We propose a stochastic generative model to represent a directed graph constructed by citations among academic papers, where nodes and directed edges represent papers with discrete publication time and citations respectively. The proposed model assumes that a citation between two papers occurs with a probability based on the type of the citing paper, the importance of cited paper, and the difference between their publication times, like the existing models. We consider the out-degrees of citing paper as its type, because, for example, survey paper cites many papers. We approximate the importance of a cited paper by its in-degrees. In our model, we adopt three functions: a logistic function for illustrating the numbers of papers published in discrete time, an inverse Gaussian probability distribution function to express the aging effect based on the difference between publication times, and an exponential distribution (or a generalized Pareto distribution) for describing the out-degree distribution. We consider that our model is a more reasonable and appropriate stochastic model than other existing models and can perform complete simulations without using original data. In this paper, we first use the Web of Science database and see the features used in our model. By using the proposed model, we can generate simulated graphs and demonstrate that they are similar to the original data concerning the in- and out-degree distributions, and node triangle participation. In addition, we analyze two other citation networks derived from physics papers in the arXiv database and verify the effectiveness of the model.

## Introduction

Scientific papers are major achievements in the academic field. Recently, the number of academic papers has increased rapidly; hence, it is necessary to evaluate their quality. Impact factor [1] and h-index [2] are well-known indicators for evaluating the quality of academic journals and authors, based on the quality of the papers. The field that studies the approach to such evaluation is called institutional research (IR), and it garners considerable interest in the academic society. In IR, analyzing the formal information of papers, such as citation structures or co-authorships, is a major topic. In this study, we are interested in elucidating the citation structure by constructing a stochastic generative model. The citation structure among papers is usually represented as a network (or a directed graph), called a citation network, where papers and citations are represented as nodes and directed edges, respectively. The analysis of citation network allows us to validate the importance of papers; for example, a paper with a large number of citations is considered important. Note that in-degree is an approximation of the “importance” of a paper, and there are other definitions for “importance” such as given in [3].

Several studies have proposed network models to grow a network, which are categorized as random graph generators. The Barabási–Albert model [4] attempted to express the growth of the Internet web pages using the well-known preferential attachment (PA) mechanism. In the web network, web pages and links correspond to nodes and edges, respectively. PA mechanism implies that a web page linked by more other web pages receives more links. It is well known that a network generated by PA exhibits the in-degree distribution, in accordance with the power law. We note that this model is similar to the Price model [5]. Although PA is proposed as a model for a web network, it has significantly influenced the analysis of citation networks. In addition to PA, the Holme–Kim model [6] introduced the triad formation (TF) mechanism because an important feature of citation networks is many appearances of triangles, i.e., connected three nodes. One TF generates more than or equal to one triangle in adding an edge. In this model, when generating edges, PA was solely performed just for the first edge. Then PA and TF were performed randomly with some probabilities. If the probability of TF is zero, the model is the same as the Barabási–Albert model. The Barabási–Albert and Holme–Kim models assume that the out-degree is constant. Later, the Wu–Holme model [7] introduced the aging effect, which considers the time difference between two papers to decide the edge generating probability. Note that this model approximates a publication time by node IDs, adopts the out-degrees of data when it simulates a network, and selects a node considering aging effects instead of in-degrees.

Krapivsky and Redner [8] note the large number of duplicates that appear in citations, which they call copies, and Simkin and Roychowdhury [9] report that the percentage of copies in scientific citations occupates 80%. Although the copy model has similarities with Holme–Kim and Wu–Holme’s TF in terms of the density of citation structures, they are not strictly equivalent, as the selection probability beta of TF is estimated to be 0.99 for scientific citations in the same field. The difference is that the copy model selects references to the target paper as candidates for copying, whereas the TF selects cited and citing papers to the target paper as candidates for connection. Leskovec et al. [10] proposed a modeling approach using the Kronecker graph, whose adjacency matrix is defined by the Kronecker product of small parameter matrices. They explained that with a few parameters, the model can imitate networks of various fields, including citation networks.

In this study, we consider a stochastic generative model for citation networks generated on discrete time. The proposed model comprises several functions expressing the number of nodes at each time, the aging effects based on the difference in publication times between citing and cited papers, and the out-degree distribution for nodes. These functions are used to grow a network based on PA and TF mechanisms. In the next section, we discuss the data obtained from the Web of Science database. Subsequently, we define our stochastic model, estimate it using data, and demonstrate the performance of the proposed model by comparing the original data with simulated results based on our model and a few previously defined models. In addition, we similarly analyze other citation networks on the arXiv database. Finally, we conclude the paper with a few remarks.

## Citation network in Web of Science

### Web of Science bibliographic database

**Fig 1:**
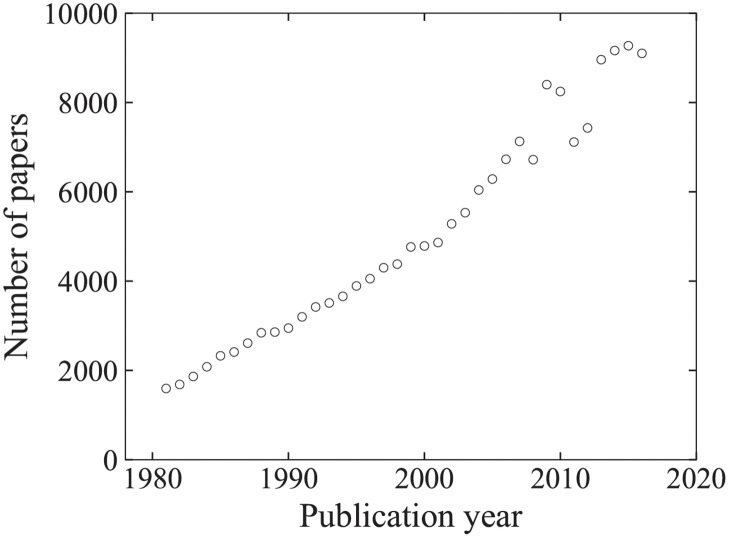
Number of papers on each publication year in WoS-Stat.

Our research was started by analyzing the citation network generated from the Web of Science (WoS), which is a famous large-scale scientific bibliographic database [11]. Each record in this database contains a title, author information, a publication time, an abstract, journal information, and a referenced paper list. Each journal belongs to several predefined subjects. Because the entire database during years 1981–2016 consists of 209.5 million papers and 1.061 billion citations and is excessively large for us to handle and consider, we focus on its subset, WoS-Stat, which is a citation network that comprises the citations between papers published in journals whose subject is associated with “Statistics and Probability.” We construct a citation network utilizing a paper identifier (ID), publication year, and reference list (list of paper IDs) for 36 years, from 1981 to 2016. WoS-Stat consists of 179483 papers and 1106622 citations. Although it includes 6411 books, we have checked that they have little effect on the following analysis. Note that the “Statistics and Probability” journals are also associated with subjects such as “Mathematics”, “Computer Science”, etc. Table 1 summarizes Top10 journals in WoS-Stat. We used publication year because the time granularity of the papers varies annually, monthly, and daily. Fig 1 presents a number of papers on each publication year in WoS-Stat. It has generally increased and saturated in recent years.

**Table 1:** TOP 10 journals in WoS-Stat.

No.	Journal	Papers
1	BIOINFORMATICS	9 268
2	COMMUNICATIONS IN STATISTICS-THEORY AND METHODS	7 559
3	STATISTICS IN MEDICINE	7 338
4	STATISTICS & PROBABILITY LETTERS	6 857
5	FUZZY SETS AND SYSTEMS	6 705
6	JOURNAL OF STATISTICAL PLANNING AND INFERENCE	5 790
7	JOURNAL OF THE AMERICAN STATISTICAL ASSOCIATION	5 045
8	COMPUTATIONAL STATISTICS & DATA ANALYSIS	4 719
9	BIOMETRICS	4 707
10	ANNALS OF STATISTICS	4 069

### Citation network

We denote the citation network using a directed graph *G* = (*V*, *E*), where a paper *i* corresponds to a node *v*_*i*_ ∈ *V*, and the citation relationship in which paper *i* cites paper *j* is represented by a directed edge (*v*_*i*_, *v*_*j*_)∈*E*. Each node *v*_*i*_ has a publication time *τ*(*v*_*i*_). We usually assume that the time is normalized as 1, 2, …, *T*.

It is evident that a paper cannot cite future papers, i.e., for an edge (*v*_*i*_, *v*_*j*_), *τ*(*v*_*i*_)≥*τ*(*v*_*j*_) should be satisfied. However, a few exceptions to this rule exist in the data. Possibly, these exceptions emerge when multiple papers are submitted in a short period of time and have citation relationships, and when there are different periods of reference processes.

It is known that the typical features of a paper include the number of papers that cite it (in-degree in graph terminology), the number of citing papers (out-degree in graph terminology). Let *A*_in_(*v*) = {*u* | (*u*, *v*) ∈ *E*} and *A*_out_(*v*) = {*u* | (*v*, *u*) ∈ *E*}, i.e., sets of the adjacent nodes of a node *v* that connects by in-coming and out-going edges. The in-degree of node *v* is defined by *d*_in_(*v*) = |*A*_in_(*v*)| and the out-degree of node *v* is defined by *d*_out_(*v*) = |*A*_out_(*v*)|, where |⋅| denotes the number of elements. We note that the out-degree of a paper depends on the type of the paper, for example, a survey paper has many citations, and a paper that analyzes data mainly has few citations in WoS-Stat.

We also consider triangle-type citation structures (node triangle participation in graph terminology) [6, 7, 10]. The number of triangles for node *v* ∈ *V* is defined by

$$\begin{array}{c} \delta (v)=|\{\left(v,v_{1},v_{2}\right)|v_{1},v_{2}\in A\left(v\right),\;\left(v_{1}\in A\left(v_{2}\right)\;\text{or}\;v_{2}\in A\left(v_{1}\right)\right)\}|. \end{array}$$

**Fig 2:**
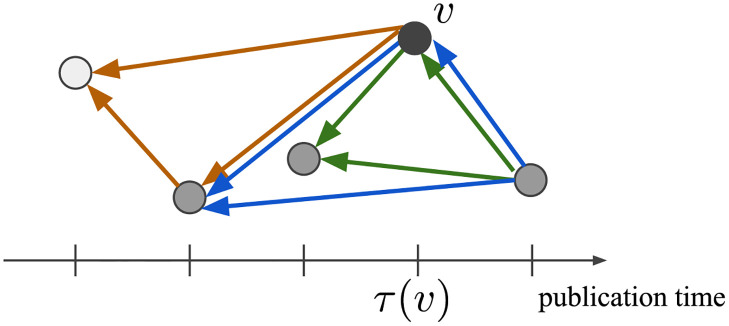
Triangles in citation network. Node *v* has 3 triangles: *δ*(*v*) = 3.

*δ*(*v*) is the number of triads (*v*, *v*_1_, *v*_2_) that consists of connected nodes (*v*_1_, *v*_2_) adjacent to *v*, ignoring the direction of edge. For example in Fig 2, node *v* has 3 triangles. Although directions of edges have a clear meaning in bibliographic contexts as citing and cited papers, we consider the number of triads for simplicity of analysis. This is called node triangle participation in [10].

In the citation network, it is known that more triangles are generated than those of the graph that uses only the preferential attachment [6], because citing and cited papers around one paper often have simultaneous citation relationships one another [6, 7].

**Fig 3:**
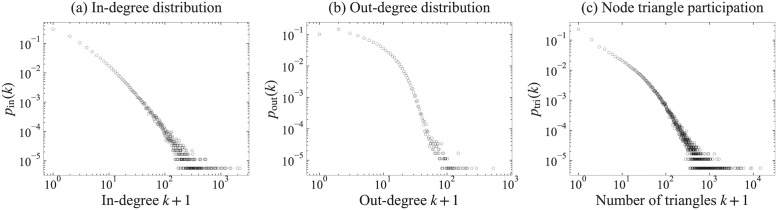
Network features in the entire WoS-Stat. The x-axis expresses (a) an in-degree *k* + 1 and (b) an out-degree *k* + 1, and (c) a number of triangles *k* + 1, while the y-axis expresses relative frequencies *p*_in_(*k*), *p*_out_(*k*) and *p*_tri_(*k*) of them.

The in- and out-degree distributions are defined as $p_{\text{in}}\left(k\right)=\frac{\left|\{v|v\in V,\;d_{\text{in}}\left(v\right)=k\}\right|}{\left|V\right|}$ and $p_{\text{out}}\left(k\right)=\frac{\left|\{v|v\in V,\;d_{\text{out}}\left(v\right)=k\}\right|}{\left|V\right|}$ with degree *k*. The node triangle participation is defined as $p_{\text{tri}}\left(k\right)=\frac{\left|\{v|v\in V,\;\delta (v)=k\}\right|}{\left|V\right|}$ with number of triangles *k*. Fig 3 illustrates the in- and out-degree distributions *p*_in_ and *p*_out_, and the node triangle participation *p*_tri_ in WoS-Stat. Note that each plot adopts the log-scale axes, and the x-axis is shifted by + 1, i.e, *x* = 1 corresponds to *k* = 0. From these figures, we can infer that they follow heavy-tailed distributions. Note that 10.2% of papers have out-degree *k* = 0; this means that these papers have no citations in “Statistics and Probability” because WoS-Stat includes citations within this field. These papers must have citations to papers in other fields or older papers before 1981, but they are outside the scope of our data.

### Features depending on time

A citation network constructed from bibliographic data has clear characteristics: older papers have fewer out-degrees, while newer papers have fewer in-degrees. If features of the entire network are modeled by considering all nodes equally, biases will appear in the modeling of the generative process. We need some corrections for features depending on time.

We define the citation age *s* by the time difference *s* = *τ*(*v*_*i*_) − *τ*(*v*_*j*_) between a citing paper *v*_*i*_ ∈ *V* and a cited paper *v*_*j*_ ∈ *A*_out_(*v*_*i*_). Then the number of citations for age *s* at time *t* is

$$\begin{array}{c} m(s,t)=|\{u|v\in V,\;\tau \left(v\right)=t,\;u\in A_{\text{out}}\left(v\right),\;\tau \left(v\right)-\tau \left(u\right)=s\}| \end{array}$$

and the citing age distribution *c*(*s*, *t*) for citing age *s* and citing time *t* is *c*(*s*, *t*) = *m*(*s*, *t*)/*n*(*t*), where *n*(*t*) = |{*v* | *v* ∈ *V*, *τ*(*v*) = *t*}| [12]. Then, we consider out-degree distribution more precisely. Out-degrees of paper *v* to age *s* is

$$\begin{array}{c} d_{\text{out}}\left(v,s\right)=|\{u|u\in A_{\text{out}}\left(v\right),\;\tau \left(v\right)-\tau \left(u\right)=s\}|. \end{array}$$

Note that 0 ≤ *s* ≤ *τ*(*v*) − 1, and *d*_out_(*v*) is given by

$$\begin{array}{c} d_{\text{out}}\left(v\right)=\sum_{s=0}^{\tau (v)-1}d_{\text{out}}\left(v,s\right) \end{array}$$

if we ignore future citations (*s* < 0). It is clear that *d*_out_(*v*) depends on *τ*(*v*) heavily, for example, *d*_out_(*v*) is near 0 if *τ*(*v*) = 1. Therefore, we correct *d*_out_(*v*) under the assumption that *c*(*s*, *t*) is almost independent with respect to time *t*. We define $d_{\text{out}}^{T}\left(v\right)$ as follows:

$$\begin{array}{c} d_{\text{out}}^{T}\left(v\right)=\sum_{s=0}^{T-1}(d_{\text{out}}\left(v,s\right)\frac{\sum_{i=0}^{T-1}c\left(i\right)}{\sum_{i=0}^{s}c\left(i\right)}) \end{array}$$

where $c\left(s\right)=\frac{1}{T-s}\sum_{t=s+1}^{T}c\left(s,t\right)$. We called $d_{\text{out}}^{T}\left(v\right)$ as the time-adjusted out-degrees and defined the time-adjusted out-degree distribution by $p_{\text{out}}^{T}\left(k\right)=\frac{\left|\{v|v\in V,\;d_{\text{out}}^{T}\left(v\right)=k\}\right|}{\left|V\right|}$ corresponding to degree *k*.

**Fig 4:**
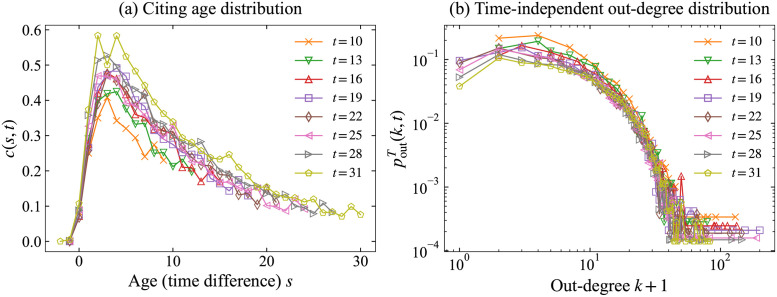
Time-adjusted characteristics in WoS-Stat. (a) Citing age distribution *c*(*s*, *t*) for citing age *s* on time *t* and (b) Time-adjusted out-degree distribution $p_{\text{out}}^{T}\left(k,t\right)$ with degree *k* for each time *t*.

Fig 4 plots the citing age distribution *c*(*s*, *t*) for citing age *s* for each time *t* and the time-adjusted out-degree distribution $p_{\text{out}}^{T}\left(k,t\right)=\frac{\left|\{v|v\in V,\;\tau \left(v\right)=t,\;d_{\text{out}}^{T}\left(v\right)=k\}\right|}{n(t)}$ for each time *t*. They are plotted for citing time *t* ∈ {10, 13, 16, 19, 22, 25, 28, 31}. It can be observed that both features *c*(*s*, *t*) and $p_{\text{out}}^{T}\left(v,t\right)$ are almost independent of time *t*. The citing age distribution is discussed in [12–14].

## Modeling of WoS-Stat network

The proposed model for a citation network comprises several components. We first assume that the expected value of the number of papers *n*(*t*) published at time *t* is approximated by the logistic function

$$\begin{array}{c} f_{n}(t|\mu_{n},\sigma_{n},\kappa_{n})=\frac{\kappa_{n}}{1+\text{exp}(-\frac{t-\mu_{n}}{\sigma_{n}})} \end{array}$$

and the considered number of papers is generated by ⎢*f*_*n*_(*t*) + *ϵ*_*n*_(*t*)⎣, where *ϵ*_*n*_(*t*) is an independent $N(0,\eta_{n}^{2})$ random variable and the floor value ⎢*x*⎣ denotes a maximum integer that does not exceed the real number *x*. We note that [12] adopted a function *f_n_*(*t* | *a*, *b*) = *a*(1 𒈒 exp(−*bt*)) for this purpose; however, we assume that it is not satisfactory, at least for WoS-Stat.

Next, we assume that the expected value of the citing age distribution *c*(*s*) for citing age (time difference) *s* is approximated by

$$\begin{array}{c} f_{c}(s|\gamma_{c},\mu_{c},\sigma_{c},\kappa_{c})=\frac{\kappa_{c}}{\sigma_{c}\sqrt{2\pi {\left(\frac{s-\mu_{c}}{\sigma_{c}}\right)}^{3}}}\text{exp}(-\frac{{\left(\frac{s-\mu_{c}}{\sigma_{c}}-\gamma_{c}\right)}^{2}}{2\gamma_{c}^{2}(\frac{s-\mu_{c}}{\sigma_{c}})}). \end{array}$$

This function is *κ*_*c*_ times the probability density function (PDF) of the inverse Gaussian distribution [15]. Note that in [7], the exponential curve is used for this purpose. However, in WoS-Stat, the citing age distribution *c*(*s*) after publication is relatively low, rapidly increases toward the peak, and then gradually decreases. Such shapes are appropriately approximated by the PDF of the inverse Gaussian distribution.

Subsequently, we assume that the time-adjusted out-degree distribution $p_{\text{out}}^{T}$ is given by the floor value of a random variable following a generalized Pareto distribution [16], whose PDF was given by

$$\begin{array}{c} f_{o}(x|\gamma_{o},\mu_{o},\sigma_{o})=\frac{1}{\sigma_{o}}{\left(1+\gamma_{o}\frac{x-\mu_{o}}{\sigma_{o}}\right)}^{-1-\frac{1}{\gamma_{o}}}. \end{array}$$

The generalized Pareto distribution is equivalent to an exponential distribution when *γ*_*o*_ = 0 and *μ*_*o*_ = 0. Since the estimation for WoS-Stat shows that these are almost zero, we will use the simpler exponential distribution, whose PDF was given by

$$f_{o}\left(x|\mu_{o},\sigma_{o}\right)=\frac{1}{\sigma_{o}}\text{exp}\left(-\frac{x-\mu_{o}}{\sigma_{o}}\right).$$

**Fig 5:**
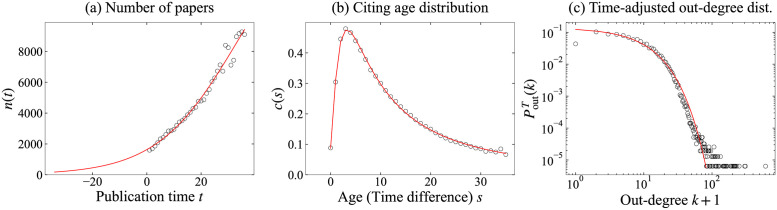
Fitted functions $\hat{f_{n}}$, $\hat{f_{c}}$, and $\hat{f_{o}}$ (red line) and WoS-Stat (black circles). (a) Number of papers: $\hat{f_{n}}$ is defined by ${\hat{\mu }}_{n}=33.263$, ${\hat{\sigma }}_{n}=14.743$, ${\hat{\kappa }}_{n}=17242.068$, and ${\hat{\eta }}_{n}=328.047$. (b) Citing age distribution: $\hat{f_{c}}$ is defined by ${\hat{\gamma }}_{c}=2.509$, ${\hat{\mu }}_{c}=-1.427$, ${\hat{\sigma }}_{c}=14.361$, and ${\hat{\kappa }}_{c}=10.191$. (c) Time-adjusted out-degree distribution: $\hat{f_{o}}$ is defined by ${\hat{\mu }}_{o}=0.000$, and ${\hat{\sigma }}_{o}=8.116$.

We estimate these functions for WoS-Stat: *n*(*t*) for *t* ∈ {1, 2, …, *T*} are used to estimate *f*_*n*_(*t*), *c*(*s*) for *s* ∈ {0, 1, …, *T* − 1} are used for *f*_*c*_(*s*), and $p_{\text{out}}^{T}$ are used for *f*_*o*_. For *f*_*n*_, We adopt the least squares method to estimate parameters and obtain estimates ${\hat{\mu }}_{n}=33.263$, ${\hat{\sigma }}_{n}=14.743$, ${\hat{\kappa }}_{n}=17242.068$, and ${\hat{\eta }}_{n}=328.047$. For *f*_*c*_, it is also estimated by the least squares method; accordingly, we obtain estimates ${\hat{\gamma }}_{c}=2.509$, ${\hat{\mu }}_{c}=-1.427$, ${\hat{\sigma }}_{c}=14.361$, and ${\hat{\kappa }}_{c}=10.191$. Although an exponential distribution variable takes continuous values, we adopt integer values $d_{\text{out}}^{T}\left(v\right)$ for each node *v* as data, to estimate parameters using the maximum likelihood method, and obtain estimates ${\hat{\mu }}_{o}=0.000$ and ${\hat{\sigma }}_{o}=8.116$ for *f*_*o*_. We estimated parameters of *f*_*c*_ and *f*_*o*_ using *c*(*s*, *t*) and $p_{\text{out}}^{T}\left(k,t\right)$ in *t* ≥ 10, which are stable and can be seen in Fig 4. Fig 5 compares fitted functions ${\hat{f}}_{n}$, ${\hat{f}}_{c}$, and ${\hat{f}}_{o}$ with real data *n*(*t*), *c*(*s*), and $p_{\text{out}}^{T}$ on WoS-Stat. We infer that these estimated functions fit well to the real network.

The last component of the model is the generating mechanism of edges. We adopt the PA and TF mechanisms considering functions *f*_*n*_, *f*_*c*_, and *f*_*o*_. Nodes at time *t* are generated according to ⎥*f*_*n*_(*t*) + *ϵ*_*n*_(*t*)⎦. Each node has out-degree generated from *f*_*o*_. We generate edges according to the combination of PA and TF, where PA and TF are performed with probability 1 − *β* and *β*, respectively. Consider that a node *v*_*i*_ is introduced to the network. In PA, *v*_*i*_ selects *v*_*j*_ ∈ *V* with probability

$$\begin{array}{c} P_{\text{PA}}\left(v_{i},v_{j}\right)\propto Im\left(v_{j}\right)\cdot f_{c}\left(\tau \left(v_{i}\right)-\tau \left(v_{j}\right)\right) \end{array}$$ (1)

where *Im*(*v*_*j*_) represents the importance of *v*_*j*_ and *f*_*c*_(*τ*(*v*_*i*_) − *τ*(*v*_*j*_)) denotes the aging effect for the time difference *τ*(*v*_*i*_) − *τ*(*v*_*j*_). In TF, *v*_*i*_ selects *v*_*k*_ ∈ *A*(*v*_*j*_) with probability

$$\begin{array}{c} P_{\text{TF}}\left(v_{i},v_{k}\right)\propto Im\left(v_{k}\right)\cdot f_{c}\left(\tau \left(v_{i}\right)-\tau \left(v_{k}\right)\right), \end{array}$$ (2)

where *v*_*j*_ is selected in the last PA and *A*(*v*_*j*_) denotes the adjacent nodes of *v*_*j*_. Subsequently, we repeat PA or TA specified times using the out-degree of *v*_*i*_. It is difficult to determine the importance of a paper. Hence, we decide to adopt the value *d*_in_(*v*) + 1 as *Im*(*v*). The verification of the proposed model will be provided by simulations in the next section.

Our edge generation mechanism combines the PA proposed by [4] (Barabási–Albert model) and TF proposed by [6] (Holme–Kim model). The Wu–Holme model [7] incorporates the edge generation that considers the change in citation ratio with the time difference, which is also called the aging effect. The PA on the proposed model considers both the importance and the aging effect with the time difference, similar to [17].

## Simulations and diagnosis of the model

### The simulation algorithm

In general, it is challenging to verify the suitability of a graph generative model. In this study, we adopt simulation experiments for this purpose. As aforementioned, the WoS-Stat network model has several components, and we executed simulation as precisely as possible, based on these components.

We first set nodes *V*′ and edges *E*′, which are initialized by ∅. We shifted the integer *t* from −*T* + 1 to *T*. Note that *V*′ and *E*′ include past time outside of given data. We added ⎥*f*_*n*_(*t*) + *ϵ*_*n*_(*t*)⎦ nodes to *V*′ at each time *t*. For *t* ≥ 1, each node *v*_*i*_ generates *k* edges using PA or TF. Here *k* = ⎥*x*⎦ and *x* were generated from *f*_*o*_(*x*). PA was first executed, and then PA or TF was executed with probabilities 1 − *β* and *β*, respectively. In our simulation based on Eq (1), PA initially selected the time difference *s* ∈ {0, 1, …, *T* − 1} with a probability proportional to *f*_*c*_(*s*), then *v*_*j*_ was selected from the subset of nodes {*v* | *v* ∈ *V*′, *τ*(*v_i_*) − *τ*(*v*) = *s*} with a probability proportional to *d*_in_(*v*_*j*_) + 1. In TF based on Eq (2), we obtained adjacent nodes *W*(*v*,_*i*_, *v_j_*, *s*) = { *v* | *v* ∈ *A*(*v_j_*), *τ*(*v_i_*) − *τ*(*v*) = *s*} \ {*v_i_*} of the node *v*_*j*_ selected at the preceding PA, for each time difference *s*. Then, we selected a time difference *s* that has a nonempty *W*(*v*_*i*_, *v*_*j*_, *s*) with a probability proportional to *f*_*c*_(*s*), and chose a node *v*_*k*_ ∈ *W*(*v*_*i*_, *v*_*j*_, *s*) with a probability proportional to *d*_in_(*v*_*k*_) + 1. When all *W*(*v*_*i*_, *v*_*j*_, *s*) were empty, PA was executed instead of TF. We skipped the edge generation when *t* was in past, i.e., *t* ≤ 0. Finally, we deleted the out-of-range nodes and edges: *V* = {*v* | *v* ∈ *V*′, 1 ≤ *τ*(*v*) ≤ *T*} and *E* = {(*v_i_*, *v_j_*) | *v_i_*, *v_j_* ∈ *V*, (*v_i_*, *v_j_*) ∈ *E*′}, respectively.

**Fig 6:**
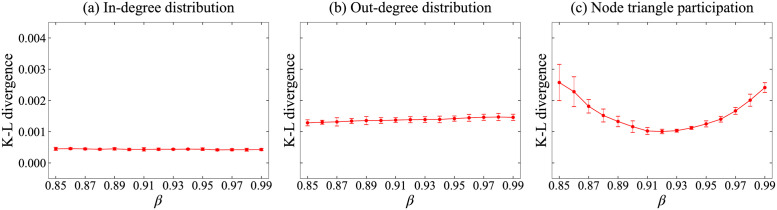
Kullback–Leibler divergences varied with *β* of the simulated data from real data WoS-Stat. (a) In-degree and (b) Out-degree distributions, and (c) Node triangle participation.

It is difficult to estimate the value of the *β* parameter; hence, we adopted simulations to determine it. We executed simulations for values of *β* from 0.85 to 0.99, with an increment of 0.01. Then we compared the Kullback–Leibler (K–L) divergence [18] between the simulated network and the original data WoS-Stat for the in- and out-degree distributions, and node triangle participation. More precisely, we compared appropriate histograms of given data to calculate K–L divergence. Fig 6 presents the mean with the approximately 95% confidence interval via ten times simulations. It can be observed that the in- and out-degree distributions are almost independent of these *β* values, and the node triangle participation takes its minimum around *β* = 0.92. So we decided to adopt *β* = 0.92. Note that *β* = 0.92 does not necessarily imply that TF is executed with a probability of 0.92 in the simulations because PA is executed at the first edge generation process.

### Simulation results

**Fig 7:**
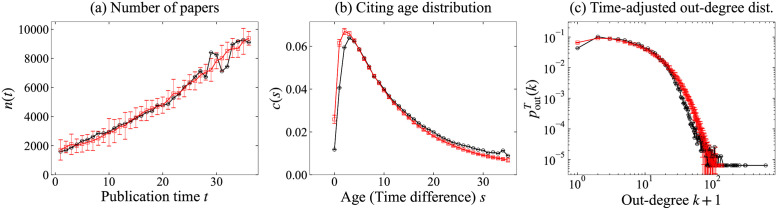
Mean with the approximately 95% confidence interval of simulation results (red squares and error bars) and real network WoS-Stat (black circles). (a) Number of papers *n*(*t*), (b) Citing age distribution $c\left(s\right)/\sum_{i=0}^{T-1}c\left(i\right)$, and (c) Time-adjusted out-degree distribution $p_{\text{out}}^{T}$.

Fig 7 presents the simulation results obtained from the simulated networks using ${\hat{f}}_{n}$, ${\hat{f}}_{c}$ and ${\hat{f}}_{o}$, and corresponding values *n*(*t*), *c*(*s*) and $p_{\text{out}}^{T}$ in WoS-Stat. Each figure presents the mean with the approximately 95% confidence interval obtained via ten times simulations. It can be inferred that all features fit well together. Note that this simulation checks the total model appropriateness, and is different from Fig 5, which checks each component separately.

We diagnosed the model fitting by visualizing network features suitable for elucidating a citation network: in- and out-degree distributions, node triangle participation, and scree plot. The scree plot shows the singular values of the graph adjacency matrix, versus their rank, using the logarithmic scale [19]. These plots were used and explained for the model validation in [10]. We compared our model with existing models: Barabási–Albert [4], Holme–Kim [6], and Wu–Holme [7]. We adopted the out-degree value 6 (the mean value of out-degrees in WoS-Stat) for each node on Barabási–Albert and Holme–Kim, which assume a constant out-degree. Holme–Kim, Wu–Holme and our models used *β* = 0.92. The Wu–Holme model requires the order of publication of papers. We adopted the sorted order by considering the paper ID and publication year in WoS-Stat. We applied the NetworkX implementations [20] of the Barabási–Albert and Holme–Kim models and implemented our model and the Wu–Holme model on this study, using the Python language with the SciPy [21] and the NetworkX [20]. We adopted the SNAP package [22] to compute network features.

**Fig 8:**
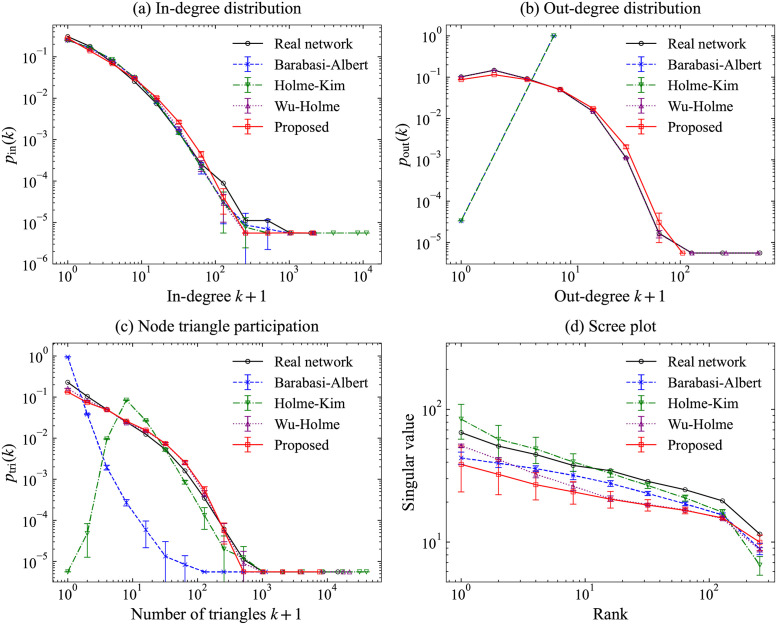
Network features in the real network WoS-Stat and simulated networks. (a) In-degree distribution, (b) Out-degree distribution, (c) Node triangle participation, and (d) Scree plot.

Fig 8 summarizes the network features generated by each model for the citation network WoS-Stat. All models succeeded in imitating the in-degrees. This may indicate that PA works well for all models. Regarding the out-degree, there are severe problems in the data generated by the Barabási–Albert and Holme–Kim models because these models assume the out-degree is a constant. The Wu–Holme model seems to fit well; however, it is natural because it uses the out-degree of the original data directly in simulations. Compared with the Wu–Holme model, the proposed model does not require the original out-degrees but adopts the estimated out-degree distribution. For the node triangle participation, the proposed model has similar results to those of the Wu–Holme model and exhibits better results than the Holme–Kim model, which does not consider the aging effect. Although scree-plot has a similar pattern among models, our model has larger differences from the data than other models.

Note that our model can produce a legitimate simulation results for considered network features.

## Citation networks in arXiv

This section shows that the proposed model also works well for two other citation networks: arXiv-HepTh and arXiv-HepPh. These citation networks are generated from papers and citations of the high-energy physics fields, hep-ph and hep-th, in the bibliographic data of arXiv [23]. We used the data available from the SNAP project [24]. The arXiv-HepTh has publication dates. Because almost all papers did not have the publication date in arXiv-HepPh, we assumed their publication months from their paper IDs. Note that arXiv-HepTh was analyzed in [7, 10, 12] and arXiv-HepPh in [10]. We analyzed data quarterly to have more than hundreds of records in one period, which have 44 and 40 time periods. Table 2 summarizes the citation networks, arXiv-HepTh and arXiv-HepPh.

**Table 2:** Summary of citation networks arXiv-HepTh and arXiv-HepPh.

Instance	Papers	Citations	Periods
arXiv-HepTh	27 770	352 285	1992/01–2002/12 (11 years, 44 quarters)
arXiv-HepPh	34 546	421 578	1993/01–2002/12 (10 years, 40 quarters)

**Fig 9:**
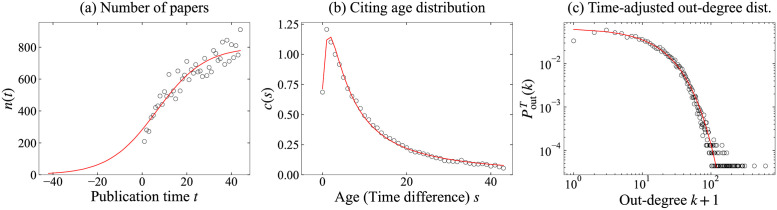
Fitted functions $\hat{f_{n}}$, $\hat{f_{c}}$, and $\hat{f_{o}}$ (red line) and arXiv-HepTh (black circles). (a) Number of papers: $\hat{f_{n}}$ is defined by ${\hat{\mu }}_{n}=6.556$, ${\hat{\sigma }}_{n}=10.779$, ${\hat{\kappa }}_{n}=802.887$, and ${\hat{\eta }}_{n}=56.445$. (b) Citing age distribution: $\hat{f_{c}}$ is defined by ${\hat{\gamma }}_{c}=4.738$, ${\hat{\mu }}_{c}=-1.527$, ${\hat{\sigma }}_{c}=9.328$, and ${\hat{\kappa }}_{c}=19.038$. (c) Time-adjusted out-degree distribution: $\hat{f_{o}}$ is defined by ${\hat{\mu }}_{o}=0.000$ and ${\hat{\sigma }}_{o}=16.164$.

**Fig 10:**
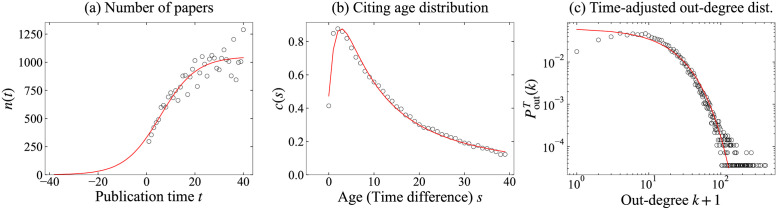
Fitted functions $\hat{f_{n}}$, $\hat{f_{c}}$, and $\hat{f_{o}}$ (red line) and arXiv-HepPh (black circles). (a) Number of papers: $\hat{f_{n}}$ is defined by ${\hat{\mu }}_{n}=5.775$, ${\hat{\sigma }}_{n}=7.416$, ${\hat{\kappa }}_{n}=1050.755$, and ${\hat{\eta }}_{n}=85.153$. (b) Citing age distribution: $\hat{f_{c}}$ is defined by ${\hat{\gamma }}_{c}=8252.979$, ${\hat{\mu }}_{c}=-2.270$, ${\hat{\sigma }}_{c}=14.985$, and ${\hat{\kappa }}_{c}=28.438$. (c) Time-adjusted out-degree distribution: $\hat{f_{o}}$ is defined by ${\hat{\mu }}_{o}=0.000$ and ${\hat{\sigma }}_{o}=16.851$.

**Fig 11:**
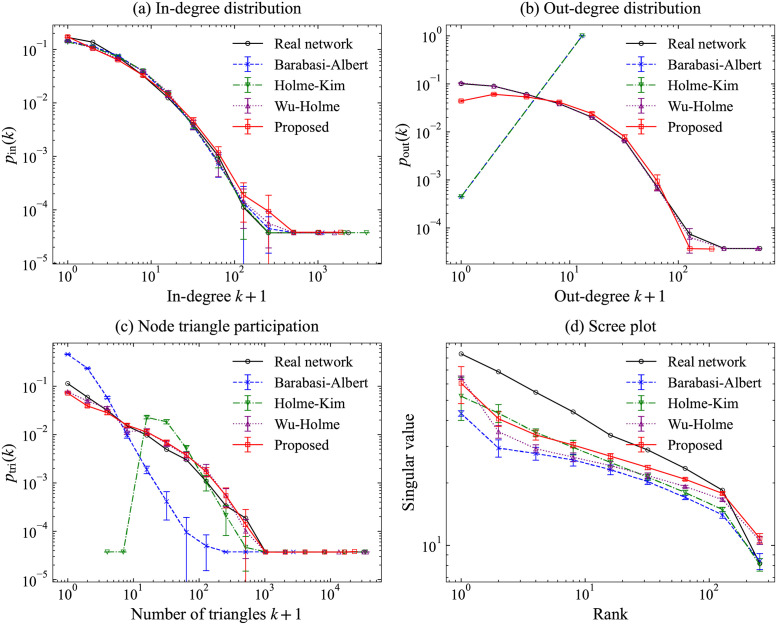
Network features in the real network arXiv-HepTh and simulated networks. (a) In-degree distribution, (b) Out-degree distribution, (c) Node triangle participation, and (d) Scree plot.

**Fig 12:**
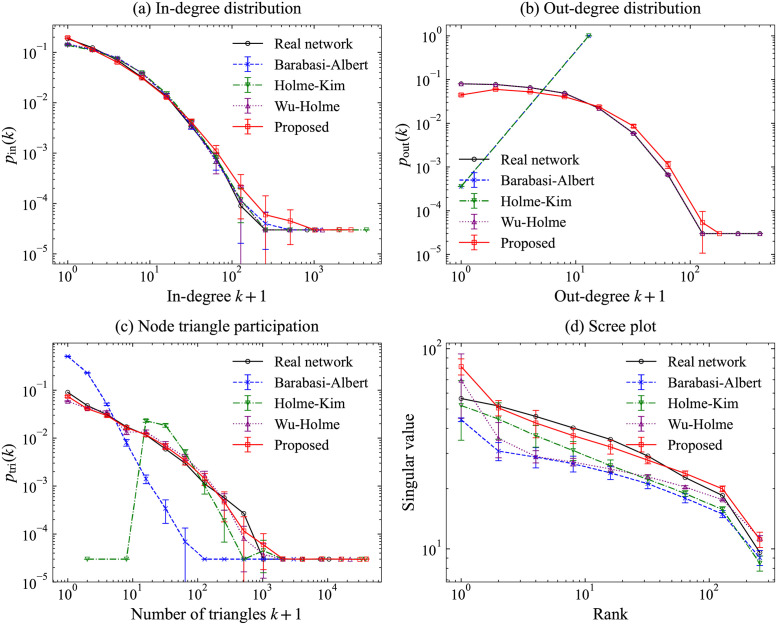
Network features in the real network arXiv-HepPh and simulated networks. (a) In-degree distribution, (b) Out-degree distribution, (c) Node triangle participation, and (d) Scree plot.

We estimated parameters of *f*_*c*_ and *f*_*o*_ with *t* ≥ 10 and compare fitted functions in arXiv-HepTh in Fig 9 and arXiv-HepPh in Fig 10. It can be deduced that they all fit well with the real networks. Network features relative to arXiv-HepTh and arXiv-HepPh are presented in Figs 11 and 12. Holme–Kim, Wu–Holme, and our models used *β* = 0.99 in both arXiv-HepTh and arXiv-HepPh, which are obtained by performing simulations similar to Fig 6 in WoS-Stat. This result for *β* is consistent with the existing model [7]. Similar to WoS-Stat case, the proposed model visually fits well for in- and out-degree distributions and node triangle participation. For the scree plot, the proposed model is confirmed to fit the same or more, compared with other models for arXiv-HepTh and arXiv-HepPh. The proposed model fits better than the Wu–Holme model, especially from the scree plot for arXiv-HepPh.

## Discussion and concluding remarks

We proposed a stochastic generative model for a graph representing a citation network. Our research motivation first came from the citation network WoS-Stat generated from the statistics and probability field in the bibliographic data from the Web of Science. We obtained the models on this network for the number of papers on publication time, the citing age distribution, and the time-adjusted out-degree distribution. In other words, we assume that their structures do not change for all publication times in the data. These assumptions are supported by the data to some extent and are required to estimate parameters accurately. However, today, situations in the academic society are changing rapidly. So the citation structure may change in the future.

We adopted three functions to define the model: a logistic function, an exponential distribution, and an inverse Gaussian probability density function. These functions were selected to approximate the data. However, it is difficult to interpret or theoretically verify their meaning. Our objective is to ensure that they are beneficial in generating similar data to the original data. Accordingly, we adopted PA and TF mechanisms. PA is employed to approximate the importance of the paper by the in-degree. We understand that the true importance of a paper is a latent variable and needs to be estimated by a significantly more complex model. We considered that the out-degree approximates the type of a paper; for example, a small out-degree indicates that the paper focuses on other fields. Because cited papers of old papers are not included in the data, they usually exhibit a small out-degree. Therefore, we considered papers focusing on other fields and old papers are in the same type. It may be problematic. In-degree and out-degree consider relationships between two nodes. Triangle considers relationships among three nodes. Hence, it is clear that our model explicitly considers the relationships among up to three nodes. We demonstrated that our model is a simple but satisfactory approximation of the graph generating process in this constraint. This constraint may explain that the scree plots of the simulated graphs tend to be relatively apart from that of original data because the scree-plot can exhibit the relationships among more than three nodes.

The important feature of the model is that the discrete-time is considered explicitly, and the discrete-time information is easy to interpret the graph structure. In addition, it enables the data generation outside of the data period, especially in past time. We can generate edges in the past and execute simulations similar to the real situation. The outside nodes and edges are discarded in the final phase of the generative algorithm, similar to the real data. This differs from other existing models [4, 6], and [7]. These models approximate the initial state with a small connected component and grow it while maintaining the connectivity. Therefore, the generated graph structure is always a connected component, unlike the proposed model. It can be observed that the proposed model is effective for other citation networks arXiv-HepTh and arXiv-HepPh. Consequently, we can expect that the proposed model provides a good approximation of general citation networks.
